# Ruptured Anterior Communicating Artery Aneurysm in an Elderly Patient With Cerebrovascular Anomalies: A Case for Palliative Management

**DOI:** 10.7759/cureus.99343

**Published:** 2025-12-15

**Authors:** Abdulrahman J Aakef, Ryan Darayseh, Aysha Al Shamsi, Hamda Al Shehhi, Khadeeja A., Abdullah O Al Ani, Saabh I Khalil

**Affiliations:** 1 Medicine, Ajman University, Ajman, ARE; 2 Medicine, Mohammed Bin Rashid University of Medicine and Health Sciences, Dubai, ARE; 3 Radiology, Sheikh Khalifa Medical City Ajman, Ajman, ARE

**Keywords:** anatomical variants, anterior communicating artery aneurysm, artery aneurysm rupture, intracranial hemorrhage (ich), neurosurgical emergency, palliative care, rupture of aneurysm

## Abstract

The anterior communicating artery is a key component of the Circle of Willis and a frequent site of intracranial aneurysm formation, particularly in elderly individuals in whom rupture carries substantial morbidity and mortality. We describe an 80-year-old hypertensive woman who presented after a sudden collapse and was found unconscious with a Glasgow Coma Scale score of 3, requiring immediate airway protection and intubation. Brain multidetector computed tomography revealed extensive acute hemorrhage involving the suprasellar region, bilateral frontal lobes, ventricles, and subdural spaces, and computed tomography angiography identified a ruptured anterior communicating artery aneurysm and a second smaller middle cerebral artery aneurysm, along with multiple cerebrovascular anomalies, including left internal carotid artery occlusion with collateral flow across the anterior communicating artery, hypoplastic carotid vessels, and posterior circulation irregularities. The combination of extensive hemorrhage, severe neurological compromise, and complex vascular anatomy rendered surgical or endovascular treatment nonviable, and the patient was transitioned to comfort-focused palliative management. This case highlights the importance of considering non-traumatic intracranial hemorrhage from aneurysmal rupture in elderly patients presenting with sudden collapse and underscores the need for urgent neuroimaging to guide timely decision-making. When imaging reveals catastrophic hemorrhage and a prognosis incompatible with meaningful recovery, early transition to palliative care is appropriate to prioritize patient comfort and dignity.

## Introduction

Anterior communicating artery (AcoA) aneurysms are the most common intracranial aneurysms, accounting for 23-40% of intracranial aneurysms and 12-15% of unruptured aneurysms [1]. They have relatively complex anatomical structures and variations. These aneurysms are clinically notable as a result of their inclination to rupture, resulting in catastrophic subarachnoid hemorrhage (SAH) with high disease burden and fatality rates [2,3]. Although the prevalence of aneurysmal rupture decreases over time with aging, the consequences often are worse and more severe in geriatric cohorts [2] due to diminished physiologic reserve, higher comorbidity burden, and reduced tolerance to complications such as early brain injury and delayed cerebral ischemia. These age-related factors limit the safety margin for aggressive intervention, thereby strengthening the rationale for discussing palliative pathways in selected geriatric patients.

In managing the surgical risk of an ACoA aneurysm rupture in an older person, aged 80 years or older, the assessment of this population presents unique clinical challenges. Variations in cerebrovascular structure due to anatomical abnormalities will also complicate the treatment planning process for these patients because they can change the regional hemodynamic flow pattern and therefore affect the formation of aneurysms and their risk for rupture [4]. Importantly, these anatomical variations also affect the feasibility and safety of intervention by limiting access, reducing visualization, and increasing procedural complexity. Given the limited efficacy of treatment interventions and the likelihood of a poor neurologic outcome, aggressive management is often not recommended in this situation [5].

## Case presentation

An 80-year-old hypertensive woman was brought to the emergency department on 12 April 2025 after being found collapsed at home, lying in vomitus and unresponsive. Her Glasgow Coma Scale (GCS) score on arrival was 3/15. Emergency responders first used a laryngeal mask airway to manage her, and then she was intubated for mechanical ventilation in the emergency room. Her initial vital signs included a temperature of 36.5 °C, heart rate (HR) of 72 beats per minute, blood pressure (BP) of 127/100 mmHg, and oxygen saturation (SpO_2_) of 99% on mechanical ventilation. She had no documented history of other chronic illnesses. Laboratory investigations revealed polymorphonuclear leukocytosis (21.14 × 10⁹/L, 93.8% neutrophils), elevated C-reactive protein (CRP) (53.4 mg/L), hyperglycemia (13.7 mmol/L), and a markedly raised troponin I (738 ng/L), which was suspected to represent neurogenic cardiac injury secondary to the severe subarachnoid hemorrhage, further underscoring the catastrophic physiological stress of the event.

 The laboratory results are summarized in Table 1. 

**Table 1:** Laboratory results of the patient on admission. The marked leukocytosis with neutrophilia was interpreted as a stress response to the acute neurologic insult rather than early infection, which is consistent with the overall catastrophic presentation.

Labs	Results	Reference Range
Temperature	36.5°C	36.1–37.9°C
Blood Pressure	127/100 mmHg	90/60–120/80 mmHg
Heart Rate	72 bpm	60–100 bpm
SpO_2_	99%	95–100%
Leukocyte	21.14 × 10⁹/L	(4.0–10) x 10⁹/L
Neutrophils	93.8%	40–70%
C-Reactive Protein	53.4 mg/L	<5 mg/L
Blood Glucose	13.7 mmol/L	<7.8 mmol/L
Troponin I	738 ng/L	<16 ng/L

**Figure 1:**
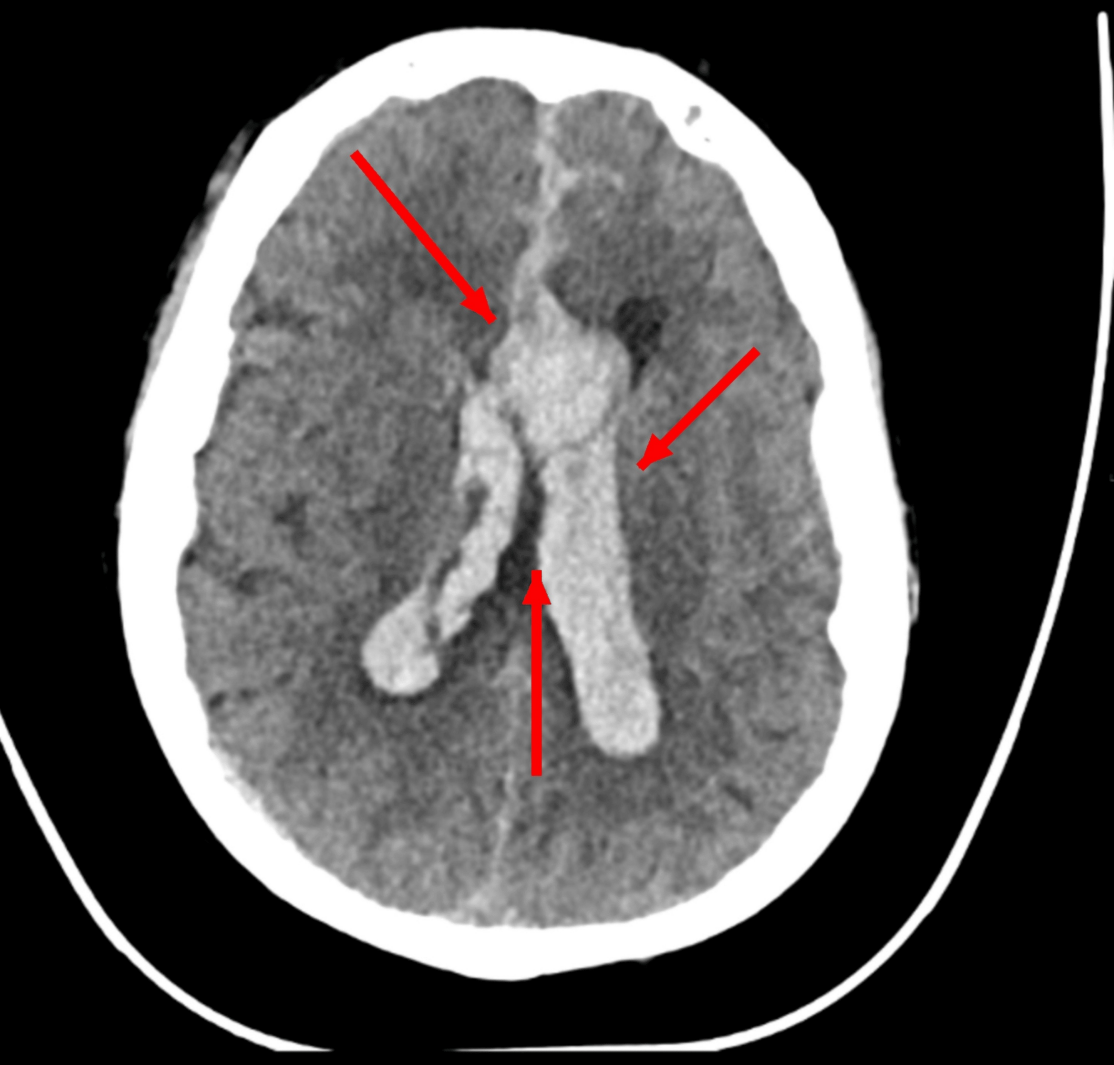
Axial section CT scan at the level of the lateral ventricle showing intra-parenchymal, subarachnoid, and intraventricular bleeding (CT scan at presentation).

An urgent non-contrast multislice CT (MDCT) of the brain demonstrated a massive acute hemorrhage centered in the suprasellar cistern, extending into the bilateral frontal lobes and anterior interhemispheric fissure, with blood layering within the lateral, third, and fourth ventricles, causing moderate hydrocephalus. Extension of blood into the foramen of Luschka and Magendie was noted. An intraparenchymal hematoma measuring approximately 34 mL was identified, along with a right-sided subdural hematoma measuring up to 4.7 mm. Basal cisterns were effaced with evidence of posterior fossa compression, suggesting early transtentorial herniation changes, which further indicated a grave neurologic prognosis. As seen in Figure 1, the axial CT scan demonstrated intraparenchymal, subarachnoid, and intraventricular bleeding.

**Figure 2:**
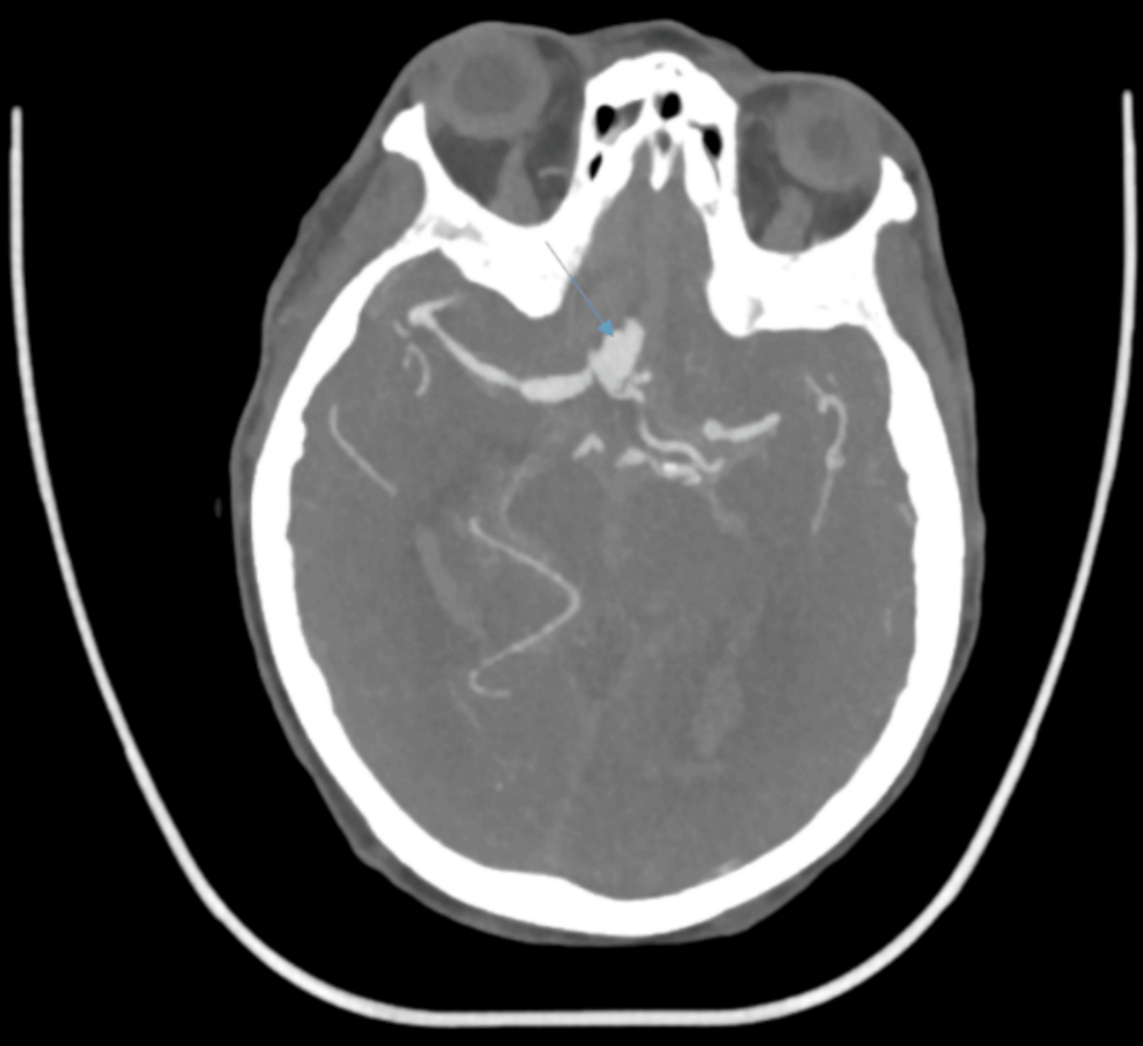
Axial section CTA showing an irregular outline of the anterior communicating artery aneurysm.

**Figure 3:**
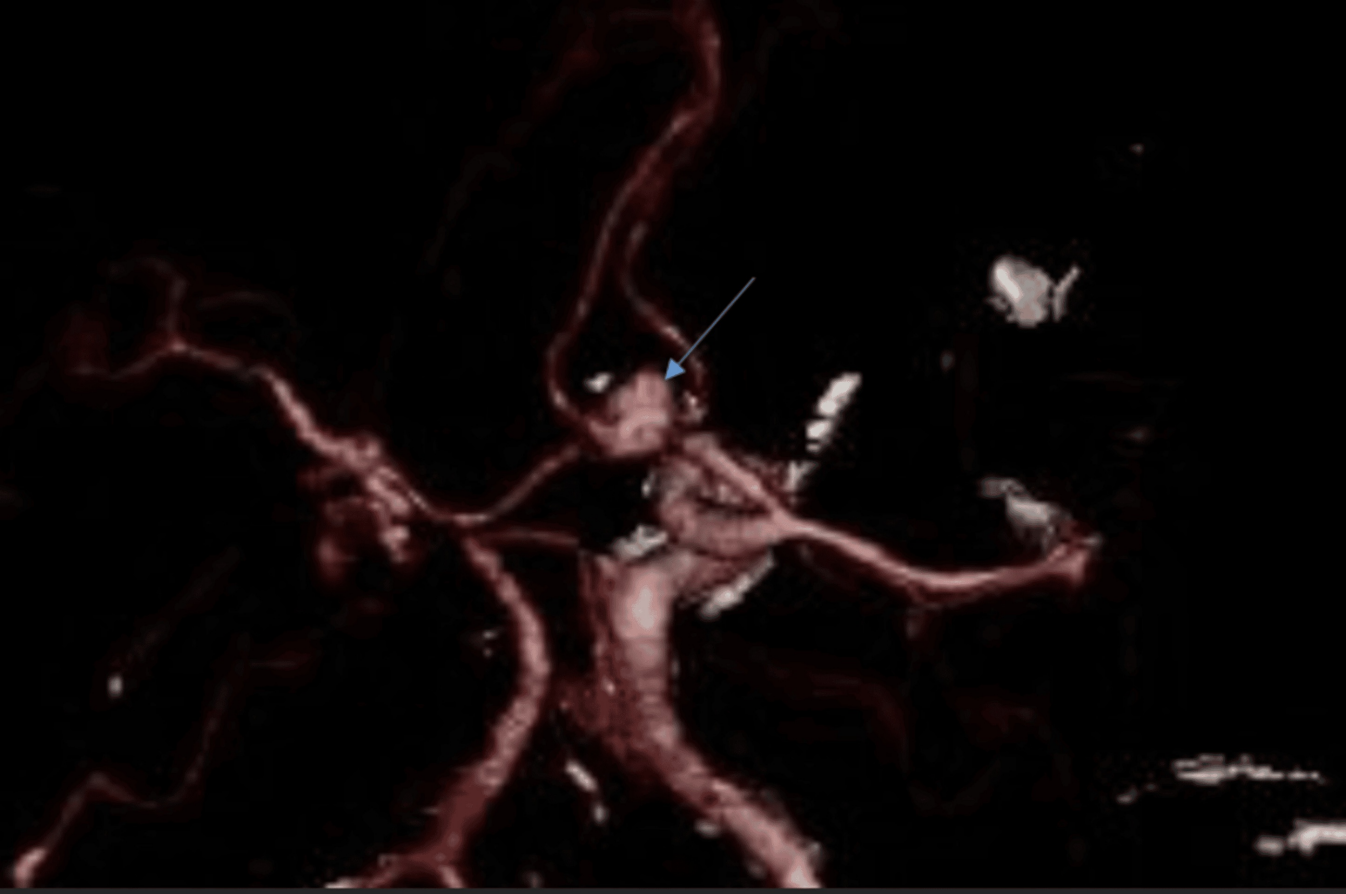
3D CTA showing the anterior communicating artery aneurysm.

A CT carotid angiogram performed the same day revealed a large ruptured anterior communicating artery (ACoA) aneurysm measuring 12.8 × 7.9 × 9.3 mm, as well as a second aneurysm at the M1 segment of the left middle cerebral artery measuring 6 × 4.5 × 4.5 mm. Additional vascular anomalies included complete occlusion of the left internal carotid artery with collateral supply maintained via the ACoA, a hypoplastic left common carotid artery terminating in the external carotid artery with a small carotid canal, stenosis of the left P1 segment with ectasia of the P2 segment, poor distal filling of the left posterior cerebral artery, and an overall abnormal vascular configuration. The reliance on collateral flow across the ACoA due to ICA occlusion likely increased local hemodynamic stress, helping explain the heightened rupture risk of the aneurysm. As shown in Figure 2, the CT angiogram revealed the irregular outline of the ruptured ACoA aneurysm. A three-dimensional reconstruction further confirmed the aneurysm morphology, as demonstrated in Figure 3. 

The patient was transferred to the intensive care unit (ICU), where neurological assessment continued to show poor outcomes with persistent, unchanged GCS score of 3/15. The right pupil is not reactive to light and is fully dilated, with a left ocular cataract. Other brainstem reflexes, including corneal and gag responses, were also absent on repeated examinations, further indicating a profoundly poor prognosis. A chest X-ray showed a correct placement of the endotracheal tube but also revealed a heart enlargement and a right pulmonary nodule. Over the following days, repeated serial neuroimaging verified an ongoing widespread supra- and infratentorial hemorrhage with intraparenchymal, intraventricular, subarachnoid, and subdural components. Intraparenchymal hematoma volume remained static, with unchanged vascular findings. No additional ischemic insults were found.

Considering the extensive hemorrhage, the poor cerebral vascular anatomy, and the patient’s severely depressed neurological status, multidisciplinary discussions with neurosurgical teams - involving remote consultation with Cleveland Clinic Abu Dhabi - determined that both surgical clipping and endovascular intervention would not alter prognosis. The patient had no documented advanced directives, and the decision for non-escalation was made in alignment with the family’s expressed preferences after thorough goals-of-care discussions. A plan of non-escalation and palliative management was agreed upon with the family, focusing on ventilatory support, stabilization of circulation, and comfort care.

Throughout her ICU stay, the patient remained comatose and reliant on mechanical ventilation. She later developed ventilator-associated pneumonia, with *Klebsiella oxytoca *growth confirmed from sputum and blood cultures. Even after administration of broad-spectrum antibiotics, her neurological prognosis did not improve. Repeated family meetings supported the shift to comfort-focused palliative care, with continued care directed toward preserving patient dignity and including family participation in end-of-life decision making. Following these discussions, life-sustaining treatment was gradually withdrawn in accordance with the family’s wishes, and the patient passed away peacefully under comfort care.

## Discussion

This case brings attention to the devastating outcomes following a rupture of an ACoA aneurysm in an elderly patient, exacerbated by numerous intracranial hemorrhage types and complicated cerebrovascular malformations. SAH secondary to an aneurysmal rupture is associated with a high risk of morbidity and mortality, with reported case fatality rates nearing 40-50% in elderly patients [6]. The patient exhibited a GCS score of 3/15, intraparenchymal, intraventricular, subdural, and subarachnoid hemorrhage, as well as obstructive hydrocephalus and posterior fossa compression - features all strongly predictive of poor outcomes. Based on her presentation, she met criteria for Hunt-Hess Grade V and WFNS Grade V, classifications associated with an extremely low-near-zero-likelihood of meaningful neurologic recovery [7,8].

The cerebrovascular diagnostic imaging outcomes were particularly remarkable. In addition to the ruptured ACoA aneurysm, the patient harbored an additional intact aneurysm at the M1 segment on the left middle cerebral artery (MCA), complete left internal carotid artery (ICA) obstruction accompanied by collateralization along the ACoA, and hypoplasia of the left common carotid artery. This diversion of flow through the ACoA created abnormal hemodynamic stress across the complex, a well-recognized mechanism that increases both aneurysm formation and rupture risk. The concurrence of multiple aneurysms, significant vessel occlusion, and atypical vascular architecture in this patient aggravated both the feasibility and safety of surgical or endovascular intervention.

Management options for aneurysmal-related SAH are impacted by patients' age, neurological grade at presentation, and radiological findings. Surgical clipping or endovascular coiling are the conventional interventions for ruptured aneurysms; however, their efficacy diminishes substantially in patients with poor-grade SAH (World Federation of Neurosurgical Societies (WFNS) Grade V) [9]. Several studies reveal that patients presenting with GCS 3 and absent brainstem reflexes have negligible chances of meaningful recovery [10]. In this case, coiling and clipping were technically unfeasible due to marked vessel hypoplasia, poor endovascular access from complete ICA occlusion, and hemodynamic instability, all of which rendered safe intervention impossible. The coexistence of an extensive hemorrhage, poor neurological status, and unfavorable vascular anatomy brought about a consensus against aggressive intervention.

The subsequent progression was further hindered by ventilator-associated pneumonia along with *K. oxytoca *bacteremia, pointing out the susceptibility of critically ill patients to hospital-acquired infections. Prolonged coma and mechanical ventilation markedly increase the risk of respiratory and bloodstream infections due to impaired airway protection, immobility, and invasive devices. Regardless of the use of broad-spectrum antibiotics, neurological recovery remained unattainable. This case, therefore, demonstrates not merely the natural history of devastating aneurysmal rupture but also the importance of palliative decision-making in neurosurgical critical care.

Significantly, the decision to undertake non-escalation of treatment was carried out through multidisciplinary deliberation and frequent family meetings and discussions. In the situation of advanced age, multiple concurrent illnesses, and a dismal outcome expectation, transitioning to supportive-focused interventions aligns with the moral principles of beneficence and non-maleficence while honoring patient dignity at the end of life. This approach also reflects accepted ethical frameworks, including the concept of medical futility and the best-interest standard, applied within a shared decision-making process involving the patient’s family.

## Conclusions

In conclusion, this case demonstrates the severe impact of a ruptured aneurysm in patients of older age with complex vascular anatomy. It highlights the importance of prompt neuroimaging, multidisciplinary evaluations, and transparent prognostic communication with the family members. It further emphasizes the crucial role of palliative care in ensuring dignity and comfort in patients for whom curative management is not feasible. Early recognition of a catastrophic prognosis may also prevent unnecessary or non-beneficial interventions, supporting more compassionate and appropriate care pathways.
